# Additive manufacturing of patient specific bolus for radiotherapy: large scale production and quality assurance

**DOI:** 10.1007/s13246-024-01385-1

**Published:** 2024-01-29

**Authors:** Deepak Basaula, Barry Hay, Mark Wright, Lisa Hall, Alan Easdon, Peter McWiggan, Adam Yeo, Elena Ungureanu, Tomas Kron

**Affiliations:** 1 Peter MacCallum Cancer Centre, Department of Physical Sciences, 305 Grattan Street, Melbourne, VIC 3000 Australia; 2 Peter MacCallum Cancer Centre, Department of Radiation Engineering, Melbourne, Australia; 3 Peter MacCallum Cancer Centre, Department of Radiation Therapy, Melbourne, Australia; 4 School of Applied Sciences, RMIT University, Melbourne, Australia; 5 Centre for Medical Radiation Physics, University of Wollongong, Wollongong, Australia; 6 Sir Peter MacCallum Department of Oncology, University of Melbourne, Melbourne, Australia

**Keywords:** Radiotherapy, Bolus, 3D printing, Quality assurance, Surface guidance

## Abstract

Bolus is commonly used to improve dose distributions in radiotherapy in particular if dose to skin must be optimised such as in breast or head and neck cancer. We are documenting four years of experience with 3D printed bolus at a large cancer centre. In addition to this we review the quality assurance (QA) program developed to support it. More than 2000 boluses were produced between Nov 2018 and Feb 2023 using fused deposition modelling (FDM) printing with polylactic acid (PLA) on up to five Raise 3D printers. Bolus is designed in the radiotherapy treatment planning system (Varian Eclipse), exported to an STL file followed by pre-processing. After checking each bolus with CT scanning initially we now produce standard quality control (QC) wedges every month and whenever a major change in printing processes occurs. A database records every bolus printed and manufacturing details. It takes about 3 days from designing the bolus in the planning system to delivering it to treatment. A ‘premium’ PLA material (Spidermaker) was found to be best in terms of homogeneity and CT number consistency (80 HU +/- 8HU). Most boluses were produced for photon beams (93.6%) with the rest used for electrons. We process about 120 kg of PLA per year with a typical bolus weighing less than 500 g and the majority of boluses 5 mm thick. Print times are proportional to bolus weight with about 24 h required for 500 g material deposited. 3D printing using FDM produces smooth and reproducible boluses. Quality control is essential but can be streamlined.

## Introduction

Bolus is commonly used to improve dose distributions in radiotherapy in particular if dose to skin must be optimised such as in breast or head and neck cancer. This applies to external beam radiotherapy both with photons [1] and electrons [2]. The introduction of Intensity Modulated Radiation Therapy (IMRT) has not diminished this demand and bolus optimisation can become part of the treatment optimisation itself [3]. In the case of electrons customised bolus has the additional advantage to allow the planner to modify the range of the electrons, thereby increasing conformity of treatment at the distal part of the treatment volume [4]. In all these scenarios customisation of bolus is an important feature of its optimal use.

Additive manufacturing, also often referred to as 3D printing, has become a widely used tool for rapid prototyping in industry. In radiotherapy it is now used for a variety of applications and research activities ranging from phantoms to patient matched devices such as immobilisation masks, brachytherapy applicators and bolus [5–7].

Given the need to customise bolus for individual patients and treatment scenarios it is not surprising that a large number of facilities have developed methods to produce 3D printed bolus [8–13]. Different methods of bolus generation are available ranging from optical scanners to creation of the bolus from a planning CT scan [14, 15]. In addition to this several companies have been established that manufacture bolus on demand [16]. 

In the present work we are documenting 50 months of experience with 3D printed bolus at a large cancer centre operating on five campuses. As bolus becomes an important part of the radiotherapy chain, quality assurance (QA) is essential [17]. As such, we report on our system of QA designed to rationalise additional work and review its effectiveness.

## Materials and methods

**Fig. 1:**
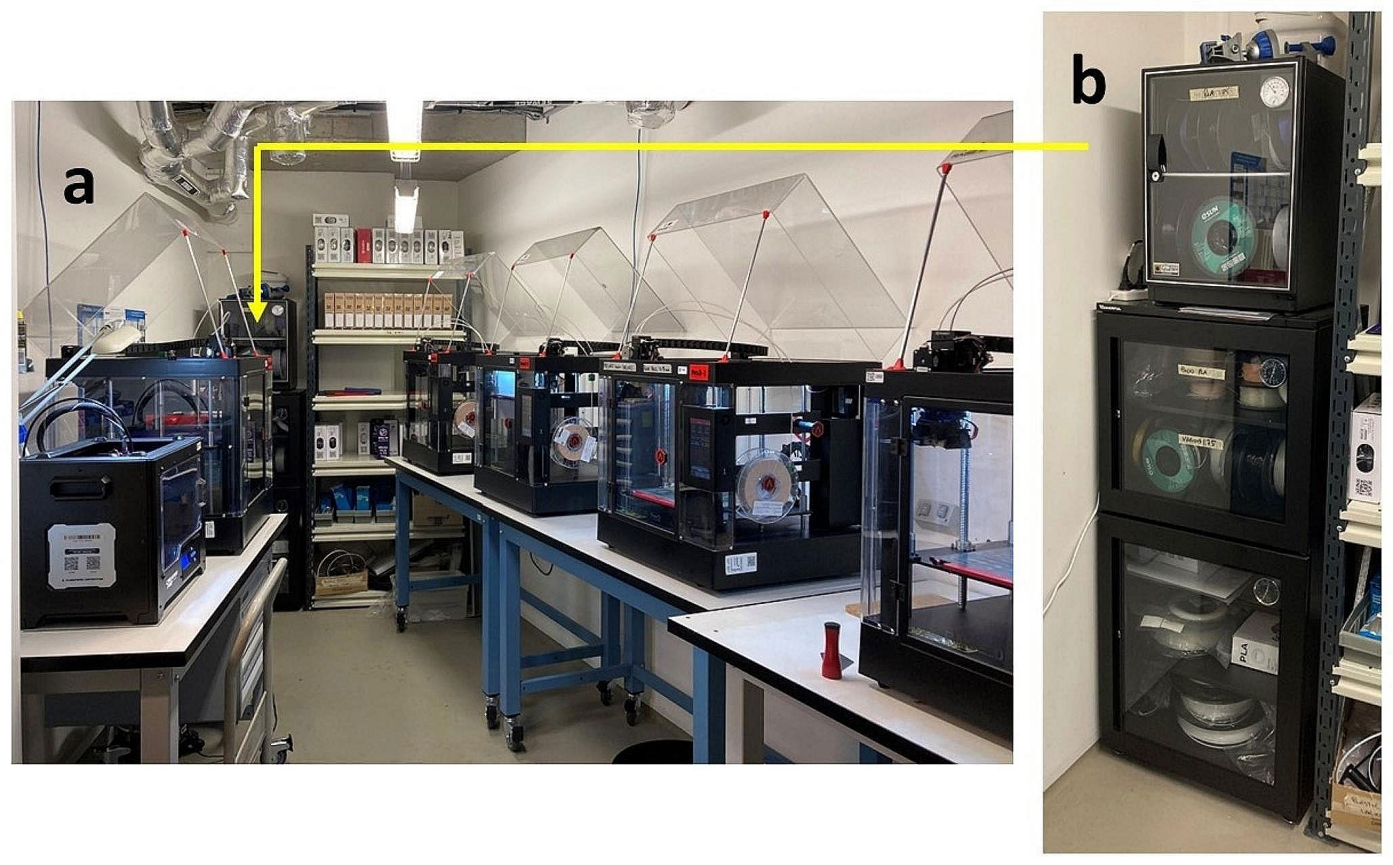
Photo of the 3D printing set-up in the mechanical workshop at Peter MacCallum Cancer Centre’s Melbourne campus. Figure 1a shows the five Raise 3D printers in use and Fig. 1b the climatised cabinet for storage of the print material

Peter MacCallum Cancer centre operates on five campuses in Victoria, Australia. Additive manufacturing was introduced in 2018 as a means to produce bolus for breast cancer patients in our largest campus at Melbourne. The process was developed by staff in the Department of Radiation Engineering/Mechanical Workshop over several months and over the following years bolus production was increased and other campuses included. In 2023, five Raise 3D printers (Raise 3D Technologies Inc., Irvine, USA) are in regular operation in the mechanical workshop as can be seen in Fig. 1.

Given the fact that several parameters determine the quality and consistency of 3D printed bolus, a set of parameters were determined *prior to* clinical printing procedure. Infill percentage was 100% with the resultant density discussed in the context of the different materials used over time. A nozzle diameter of 0.4 mm was chosen with extruder temperature 210 C, print speed 60 mm/sec and an extrusion width of 0.48 mm. Layer height was chosen as 0.2 mm. Two outline perimeters were used. Other print parameters are provided in the context of different materials used in the results section.

Raise 3D is an industrial grade Fused Deposition Modelling (FDM) printer. Several materials from different manufacturers, all polylactic acid (PLA) based were tested over the years. Criteria for selection of materials were.

- Cost and availability.
- Smoothness of printed surface.
- Lack of air inclusions in the print.
- Accuracy and reproducibility of printed dimensions.
- Colour and texture to be compatible with optical surface guidance (Align RT, Vision RT, London, UK).
- CT number and its reproducibility.

**Fig. 2:**
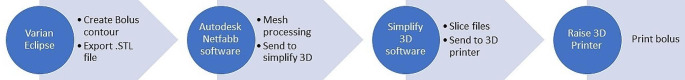
Workflow for production of bolus from Eclipse Treatment Planning System

Figure 2 shows the workflow for bolus manufacture which commences at the radiotherapy treatment planning system (Varian Eclipse, Varian Medical Systems, Palo Alto, USA) where the bolus is created on the patient’s CT scan. The bolus can be exported as a STL file that is imported into Netfabb software (Autodesk company, San Francisco, USA) and repairs, mesh re-manipulation and smoothing of the bolus contour performed. If desired at this stage also patient identification is created that can be engraved into the printed bolus to ensure the right bolus is used for every patient. Simplify3D (Cincinnati Ohio, USA) software version 5.1.2 was used to slice the 3D object and prepare it for 3D printing.

**Fig. 3:**
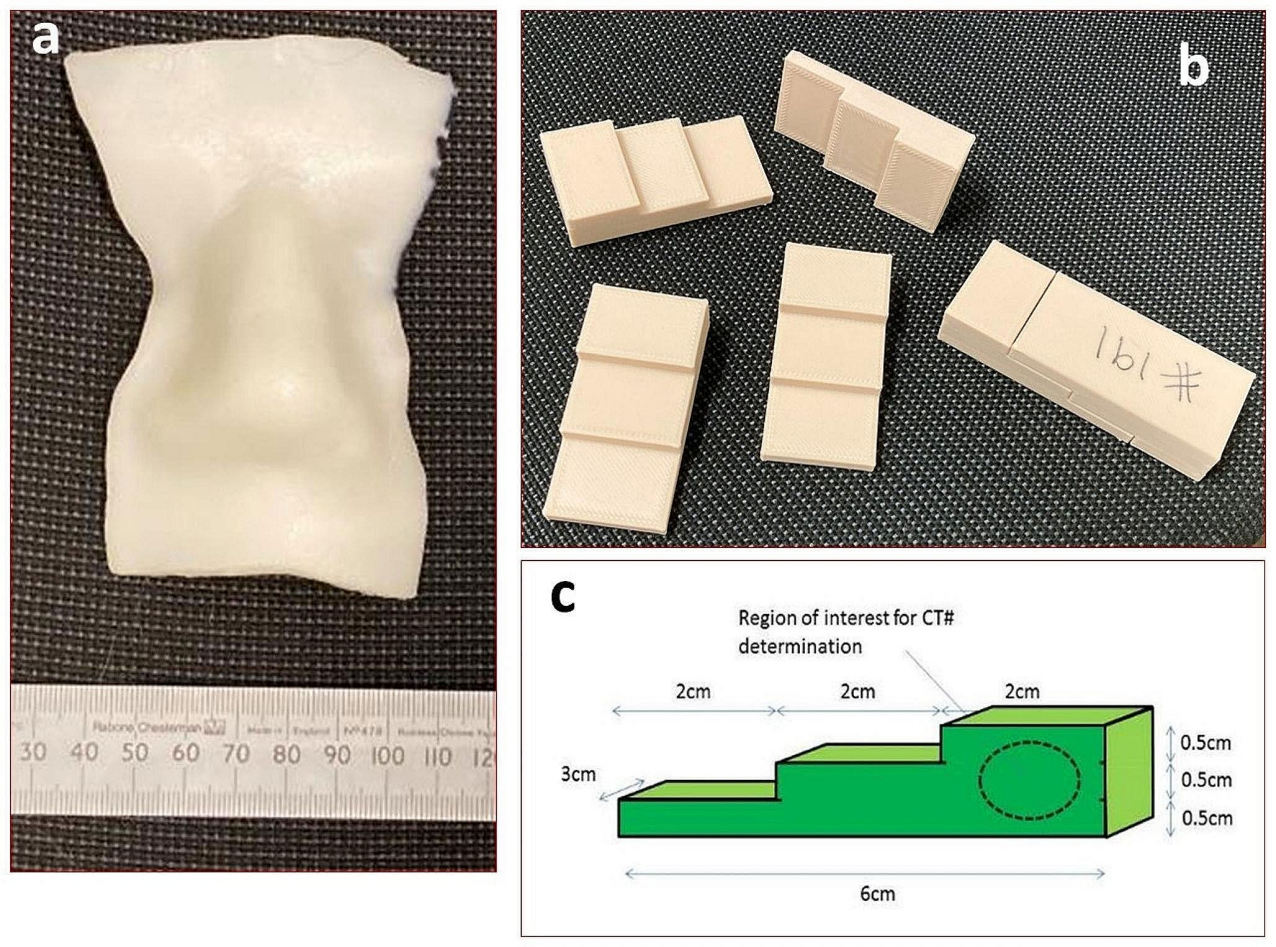
Quality Control for 3D printed bolus. **a** PLA bolus; **b** six QC wedges; **c** schematic drawing of the QC wedge with dimension. CT numbers are measured in the thick part of the wedge

Since November 2018 every bolus created is recorded in a spreadsheet (MS Excel, Microsoft, Redmond, USA) with attributes such as material used, thickness, type of bolus, weight and time required for the print. Pull down menus were created where possible to make data extraction easier. Not all fields were filled in for all boluses in particular in the early days of the spreadsheet. Also any QA activities pertaining to the bolus are recorded. Initially each bolus was CT scanned and thickness, homogeneity of material and CT number were checked. Typical tolerances were +/- 1 mm for photon bolus and 0.5 mm for electron bolus with HU numbers across the bolus required to be within +/- 100HU for photons and +/- 50HU for electrons. After checking each bolus with CT scanning for 50 samples a new and separate QA process was introduced in December 2018 [18]. As shown in Fig. 3, a three-step wedge was designed for on-going verification process to ensure consistency and quality of 3D-print boluses. The three step thicknesses mimic common bolus thicknesses (5, 10 and 15 mm) and the physical dimensions of the wedge can be easily verified using a micrometre or measurements on the CT image. Bolus density and its uniformity are assessed in terms of Hounsfield Unit (HU) from the CT scans of QC wedges, which are printed once a month or each time a new material is introduced, a new printer comes on line, software upgrades are implemented or processes are changed. In addition to the HU number and its variability the thicknesses and length of the wedge is measured using micro-callipers.

Quality control is reliant on a CT scan (Philips Brilliance 16 slice wide bore). As for most radiotherapy planning scans, 140kVp was used for the QC scan. The bolus or three step wedge was initially scanned either in air or submerged in water, which provides a better environment for HU number assessment in particular for thinner bolus. Nowadays all bolus is CT scanned in water.

Bolus was taken into consideration in the dose calculation using Acuros XB for X-rays and electron Monte Carlo for electrons (dose to medium) by assigning it a predefined CT number with a tolerance. The predefined CT number and tolerance changed over time as the processes improved and different materials became available. For electrons once each bolus is printed, a CT scan is acquired separately to record the thickness and CT number prior to clinical use.

## Results

**Fig. 4:**
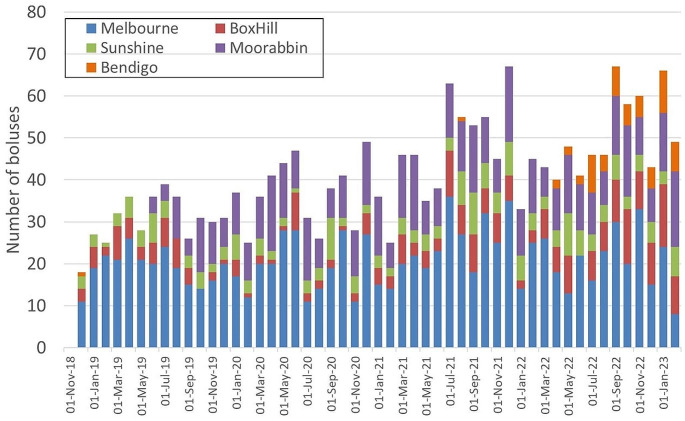
Number of boluses printed for different campuses at Peter Mac. The difference in workload explains the majority of the differences in the campuses with Bendigo, Box Hill and Sunshine having 2 linacs each, while Moorabbin has 4 and Melbourne campus 6

Between Nov 8, 2018 to Feb 28, 2023 a total of 2089 boluses were printed equating to nearly 500 boluses per year. Figure 4 shows the production of bolus with time. While there is an increase across all campuses (r^2^ = 0.523), production also increases as new campuses come on line. Once the last campus came fully on-line in July 2022, the demand per megavoltage treatment unit was fairly stable with the average number of boluses per month per linear accelerator being 3.3 +/- 1 (compare Fig. 4).

Of all boluses over the four years 93.6% were designed for photon treatments (mostly VMAT) and 6.3% for electrons. One bolus was created for a kV treatment using a superficial radiotherapy unit.

This is commensurate as the majority of patients who had a printed bolus at all five Peter MacCallum Cancer Centre sites were Head and Neck cancer treatments (*n* = 1400) with Breast/skin (*n* = 674) being second (some not recorded). The distribution of bolus thickness was 85.8% being 5 mm and 12.6% 10 mm. Thirty-four boluses had variable thickness between 5 and 15 mm mostly used for Head and Neck radiotherapy.

**Fig. 5:**
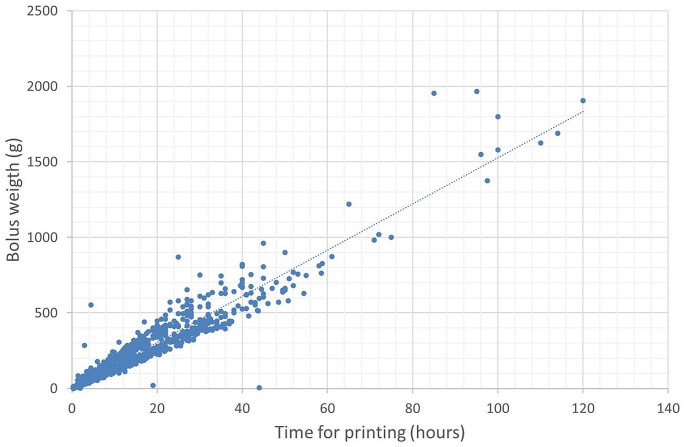
Relation between bolus weight and printing time

**Fig. 6:**
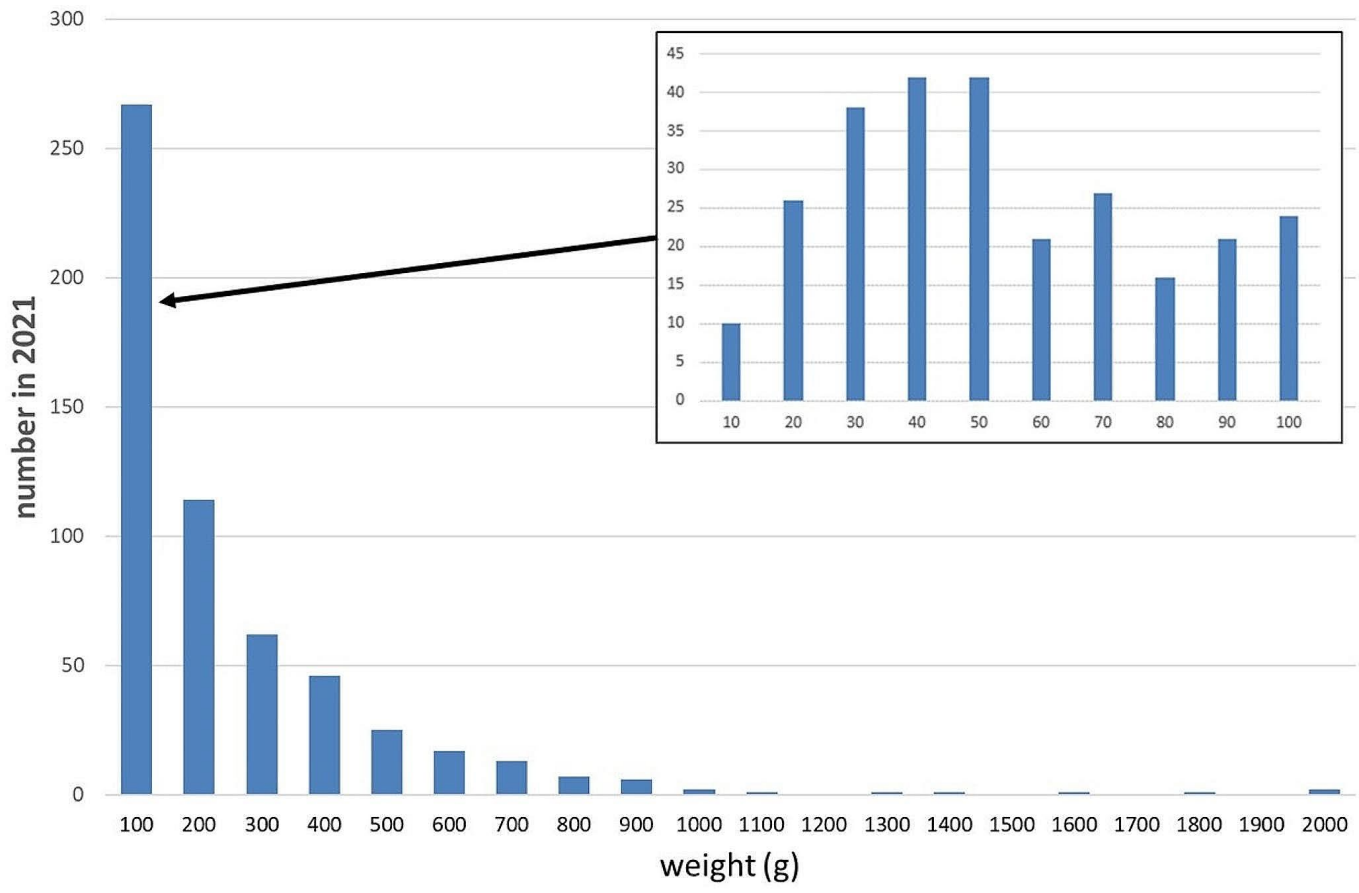
Distribution of boluses with weight in 2021

The print time increases with bolus weight as can be seen in Fig. 5. Bolus was created over the four years with weights ranging up to 1966 g requiring print times of up to 5 days. Figure 6 shows the number of boluses within a given weight range in 2021. For larger boluses that do not fit onto the print bed the print was split into two parts which were afterwards adhered together. All Raise 3D printers have a restart feature in the event of a power failure which was particularly important for long prints where power failures during the printing are more likely.

No changes of bolus weight distribution were observed over time. Figure 5 shows the distribution of boluses according to weight in 2021.

Including the post-processing of the finished bolus, QA and the other steps shown in Fig. 2, it takes typically about 3 to 4 days for the bolus from design in the planning system to the bolus being available for treatment. This makes it important to use a temporary bolus for the patient while waiting for the 3D printed bolus for example if the patient is urgently commencing treatment or if the overall printer capacity is exceeded. In this case, superflab which has a HU number of 150 is used. Provided the number of fractions with the temporary bolus is less than three or 10% of the overall number of fractions (whichever is smaller) the HU used in the plan for the 3D printed bolus is used.

Over the four years covered by this study several materials from different manufacturers were used for bolus production. Table 1 lists the materials which were utilised for patient matched bolus sorted in order of the time they were first used. Also listed are the results of the HU number measurements for bolus (initially all boluses and later for electrons only) and the QA wedges that were increasingly used over time. One can see that the premium material used now (Spidermaker PLA, Pan Asian Plastcis Corporation, Taiwan) results in both better HU number reproducibility as well as better match to the HU number of the boluses which were scanned. The material also results in smooth surfaces of the print and air bubble inclusion is rare. As such it was decided to accept the higher material cost. The nominal HU used in treatment planning for the Spidermaker PLA material is 100 with tolerances of 40 to 160HU for MV photon and 70 to 130HU for electron treatments.

**Table 1:** CT number measured for selected boluses and all QA wedges in the study period (140kVp). SD = single standard deviation; n = number of measurements

	patients			QC wedge		
3D print material	CT#	SD	n	CT#	SD	n
PLA	117	20	36			
PLA - E-sun	113	18	14			
PLA - E-sun (Natural)	123	18	23	130	10	72
PLA+ - E-sun (Beige)	132	8	2	143	29	6
PLA+ - E-sun (Skin Tone)	158	27	9	119	10	2
PLA - Aurarum	100	na	1	148	16	34
PLA - Spidermaker (Fair skin)	80	9	21	78	7	57
total			106			171

**Fig. 7:**
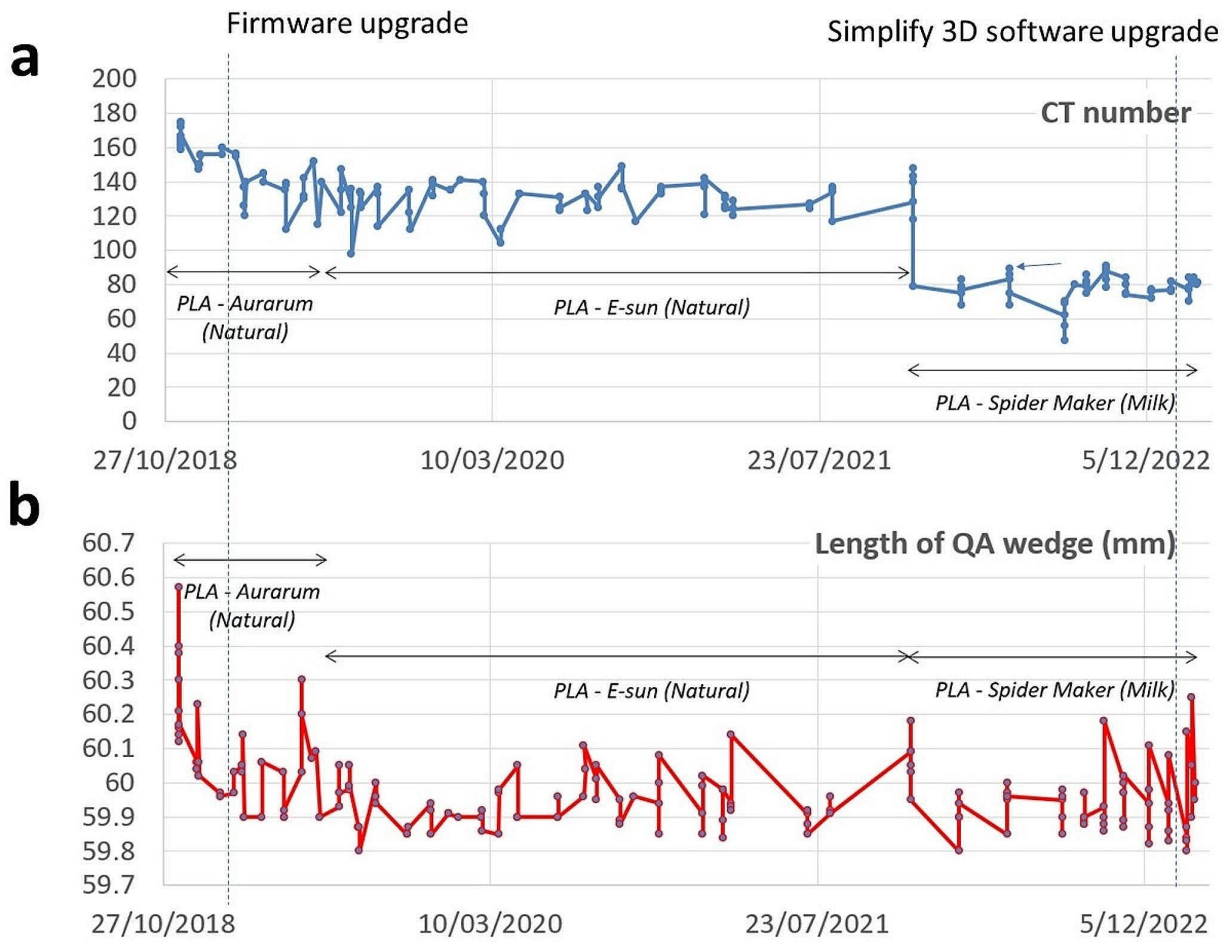
Variation of two QC parameters, CT number (Fig. 7a) and length of the printed QC sample (Fig. 7b) as a function of time. Three different materials were used over three years and the timing of two software/firmware changes is also indicated

Figure 7 shows results for CT numbers and the length of the 3D printed QC wedge for three selected materials. Also two events (firmware upgrade and simplify 3D software upgrade) are highlighted.

In addition to reproducibility the actual thickness and its reproducibility of the QA wedges was considered in material selection. Table 2 shows the thickness of the material in the three most commonly tested materials assessed for the different steps of the wedge. In addition to this length and weight of the printed sample was recorded and is shown in the table. The Spidermaker PLA shows a high quality and reproducibility of the prints. In general 3D printing was performed with an extrusion multiplier of 1 but if the HU value was considered not adequate or less dense areas were detected in the scan, the extrusion multiplier was increased to 1.02 (2% over nominal extrusion which can help to avoid unintended low density areas).

**Table 2:** Thicknesses of the QC wedge for the three most common materials. SD = single standard deviation

	PLA– Aurarum (*n* = 34)		PLA - E-sun (Natural) (*n* = 72)		PLA - Spidermaker (Fair skin) (*n* = 57)	
	Thickness (mm)	SD (mm)	Thickness (mm)	SD (mm)	Thickness (mm)	SD (mm)
5 mm step	5.1	0.1	5.1	0.1	5.0	0.1
10 mm step	10.2	0.2	10.1	0.1	10.0	0.1
15 mm step	15.2	0.2	15.2	0.1	15.0	0.2
Length (mm)	60.1	0.2	60.0	0.1	60.0	0.1
Weight (g)	22.5	0.6	21.6	1.0	20.7	0.5

**Fig. 8:**
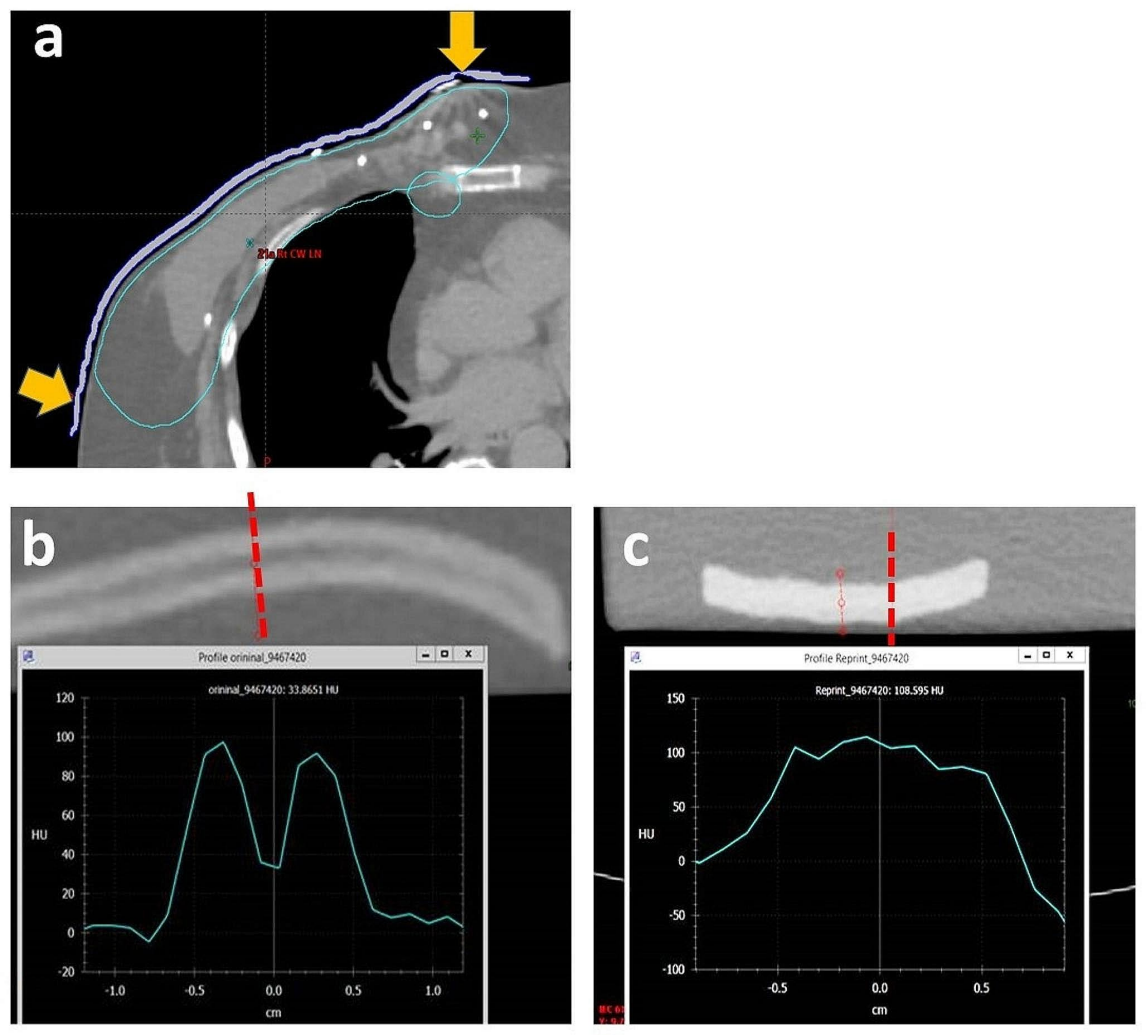
Examples of failure mode, highlighting the importance of commissioning process and staff training: **a** user error on contouring 3 mm thin bolus structure to produce 3D print input file (RT structure) in context of breast radiotherapy, **b** a pair of good and bad examples for 1 cm bolus with and without internal air-gap, respectively

One noticeable failure mode for accurate 3D-printing is the creation of thin boluses with thickness of less than 5 mm. An example of such failure is shown in Fig. 8a for breast bolus, related to contouring and associated printing procedure. Another example of failure mode is low density in the middle of bolus, as shown in Fig. 8b and c. Any bolus which is identified as deficient is reprinted after checking mesh pre-processing. At times it may also be necessary to revisit the bolus contours in the planning system to simplify them. The patient is treated temporarily with a conventional bolus (superflab) if she/he needs to progress to treatment urgently.

**Fig. 9:**
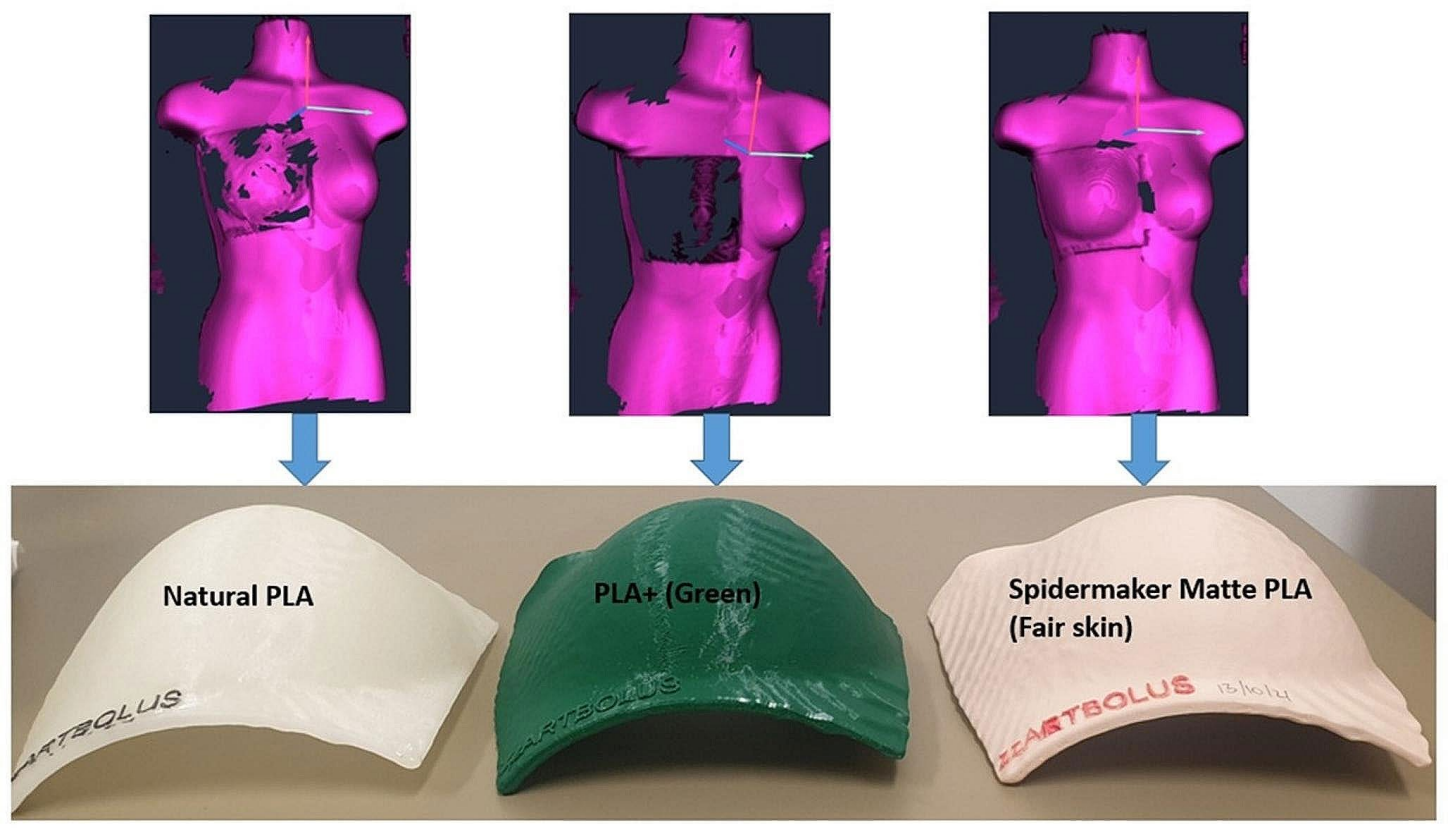
Visibility of three different PLA materials Esun Natural PLA, Esun PLA+ (Green) and Spidermaker Matte-PLA, fair skin) in optical surface guidance (AlignRT). Shown are the images of an anthropomorphic phantom with bolus for the left breast as well as the boluses used. The dark region on PLA+ (green) indicates the system’s inability to reconstruct the surface due to lack of optical signal. The boluses also show the labels that can be incorporated into the print process

Another important consideration in the choice of bolus material is the compatibility with the optical surface guidance system that was introduced in 2021 in our department [19, 20]. Figure 9 shows three different materials and their appearance in the surface guidance. The Spidermaker PLA material which features a smooth matt surface of Caucasian skin colour performed best amongst tested materials as can be seen in the figure.

## Discussion

Utilising additive manufacturing for individualised patient matched bolus creation has been demonstrated to work well resulting in a significantly better patient fit than other bolus materials [21–23]. Feedback from treatment staff regarding the ease and speed of suing 3D printed bolus has been positive. A significant amount of development work was required by the mechanical engineering staff in collaboration with radiation therapy and medical physics colleagues. Access to CT scanning and meticulous documentation of all modifications made to the process proved essential.

In Australia, patient matched devices such as bolus, are covered under medical devices and regulated by the Therapeutic Goods Administration (TGA). A clear description of the process of bolus production and quality assurance program are helpful in this process.

The success of the program can be seen in Fig. 4 which shows that once 3D printed bolus was available, the campuses that had access to it utilised it immediately. Over time all campuses at Peter MacCallum Cancer Centre commenced to use the services and the number of boluses printed for each campus monthly reflect broadly the relative number of patients treated there. Over time also the number of printers required to provide the service has increased to five.

We process about 120 kg of PLA per year and are currently investigating the potential to recycle the material. As the logistics of testing 500 boluses per year is complex and could delay the delivery of bolus for patient treatment, we developed a QC process based on regularly printed test objects as can be seen in Fig. 3. This standardised process not only makes QC faster but also allows easier tracking of material or printer changes that could affect bolus quality. As not all materials from all manufacturers are equally suited [16] and print parameters can affect the bolus [8], regular monitoring of the print quality both by mechanical and imaging is essential. The use of a ‘premium’ material at about double the cost of the cheapest PLA on the market has helped with consistency of thickness and density as well as yielded smoother surfaces.

Many radiotherapy departments now use additive manufacturing for creation of patient matched boluses [9, 17, 24]. While our practice is mostly aimed at ensuring adequate skin dose, in principle 3D printing can be used to also optimise the dose to deeper structures such as lung and heart [13]. While the majority of our bolus production goes towards improvement of photon treatments, the accuracy of bolus manufacturing in electron radiotherapy is possibly even more important [2, 10, 24]. It may also be used for improvement of dose distributions in non-pencil beam proton radiotherapy [25, 26].

Our practice utilises the planning CT scan for design of bolus. This is convenient and allows the selection of the best thickness based on the actual treatment plan. The ability of most modern treatment planning systems to export bolus in a file suitable for manufacturing is helpful even if these files require some additional manipulation (smoothing, orientation) before they can be used for printing. While other, in particular optical methods to create bolus may be attractive [14, 15, 23], it is the streamlined process afforded by the CT based bolus creation and the separation of the QA process from the patient bolus that has made the 3D printed bolus production in our centre such as busy and successful service.

Failure modes resulting in inaccuracy of printing thin bolus can be attributed mainly to two factors: i) finite contouring resolution along the curvature of patient skin and inability to achieve uniform HU including shell thickness, which becomes a considerable portion of when bolus thickness becomes less than 5 mm. In this context, we have adopted a minimum set of recommendations for additive manufacturing of patient specific bolus from planning CT scans:

- HU number can be assigned to a nominal value that depends on the material. Assessing this number in bolus in air and submerged in a water bath provides a good representation of the actual HU number.
- The tolerance of HU number may be different for MV photon treatments and electrons/kV photons where small variations in density have a greater effect on dosimetry. We assess each bolus used for electrons but perform QA on test samples for photons only.
- After each change in material and/or procedures the first boluses are measured for all treatments (including MV photons).
- The accuracy of bolus thickness for photons must be within +/- 1 mm and for electrons within +/- 0.5 mm.
- Minimum recommended bolus thickness 5 mm.
- Air gaps to skin can be assessed in MV photon beams from Cone Beam CT and should be smaller than 2 mm.

## Conclusion

After significant development work, 3D printing using FDM produces smooth and reproducible boluses. They are now used widely for breast and head and neck radiotherapy in our department. Quality control is essential but can be streamlined.
